# Cryo-EM structures of the full-length human KCC2 and KCC3 cation-chloride cotransporters

**DOI:** 10.1038/s41422-020-00437-x

**Published:** 2020-11-16

**Authors:** Ximin Chi, Xiaorong Li, Yun Chen, Yuanyuan Zhang, Qiang Su, Qiang Zhou

**Affiliations:** 1 Westlake Laboratory of Life Sciences and Biomedicine, Key Laboratory of Structural Biology of Zhejiang Province, School of Life Sciences, Westlake University, Hangzhou, Zhejiang 310024 China; 2 Institute of Biology, Westlake Institute for Advanced Study, Hangzhou, Zhejiang 310024 China; 3 School of Life Sciences, Zhejiang University, Hangzhou, Zhejiang 310058 China

**Keywords:** Molecular biology, Cryoelectron microscopy

Dear Editor,

The electroneutral K^+^-Cl^−^ cotransporters (KCCs) play fundamental roles in trans-epithelial transport, cell volume regulation, and neuronal excitability.^1^ The neuronal-specific KCC2 (SLC12A5) and glial-expressed KCC3 (SLC12A6) are essential for normal development and function of the nervous system. Mutations in KCC2 have been linked with human idiopathic generalized epilepsy^2^ and schizophrenia.^3^ KCC3-dependent Cl^−^ transport across the choroid plexus epithelia is involved in the production of cerebrospinal fluid.^4^ Here, we present single particle cryo-electron microscopy (cryo-EM) structures of the full-length human KCC2 and KCC3 at overall resolution of 3.2 and 3.3 Å, respectively (Fig. 1a, b; Supplementary information, Figs. S1, S2 and Table S1). The structures of KCC2 and KCC3 are very similar to each other. For simplicity, we mainly focused on KCC3 for structural analysis. KCC3 exists as a dimer in domain-swapping conformation (Fig. 1a). A marked difference is observed in the C-terminal domain (CTD) compared with *Danio rerio* Na^+^-K^+^ Cl^−^ cotransporter 1 (*Dr*NKCC1), which rotates ~70° clockwise as seen from cytosolic side when transmembrane (TM) domains aligned together (Supplementary information, Fig. S3a). The TM domain and CTD are connected through a pair of scissor helices (Fig. 1b), which separate the two TM domains from different protomers resulting in a unique “Central Cleft” between TM domains at the cytosolic side (Fig. 1b). The extracellular domain (ECD) of the two protomers are placed in distance (Fig. 1b). Both TM domains, scissor helices and CTD are involved in dimerization of the transporter. The TM domain of KCC3 is captured in an inward-facing conformation, sharing the LeuT-fold as the members of the amino acids, polyamines and organocations (APC) superfamily (Fig. 1b), with TMs 3–5 and 8–10 forming the scaffold domain and TMs 1, 2, 6 and 7 constituting the bundle domain (Fig. 1c). Three ion-like densities are found in the unwound region of TM1 and TM6 in KCC3 (Supplementary information, Fig. S3b, left), to which we assigned two Cl^−^ ions and one K^+^ ion with the consideration of both coordination environment and structural similarity with KCC1. In the cryo-EM map of KCC2, the densities for K^+^ and one of Cl^−^ ions are absent. Instead there is a weaker density similar to that is observed in KCC1 purified in NaCl condition^5^ (Supplementary information, Fig. S3b, right). Amino acid substitution of tyrosine (Y407 in KCC2) to phenylalanine (F441 in KCC3) causes subtle change in the ion binding site, which might provide a possible explanation for the ion coordination differences between KCC2 and KCC3 (Supplementary information, Fig. S3c).

**Fig. 1:**
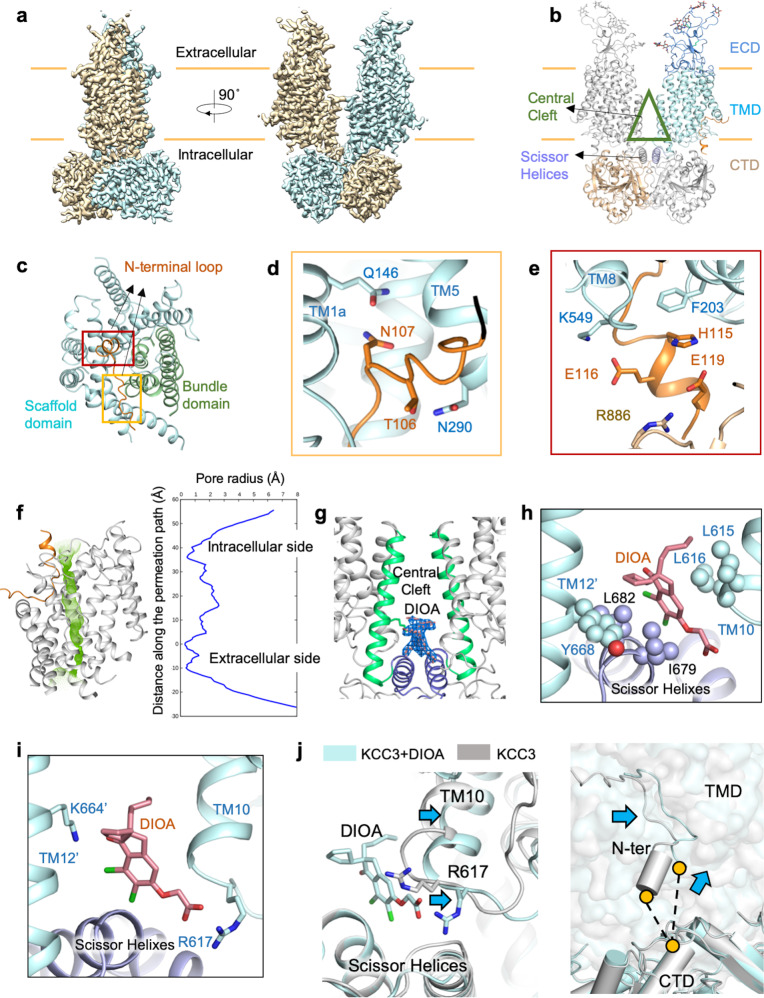
Cryo-EM Structure of human KCC3. **a** Cryo-EM map of full-length human KCC3, generated by merging maps which are refined on TM/ECD and CTD, respectively. **b** The structure model of human KCC3 colored by domains. Carbohydrate chains were shown in sticks. Unless otherwise indicated, the same domain color scheme is applied to all figures. **c** N-terminal loop locates in the cytosolic entry of the transport path of KCC3, between the bundle domain and Scaffold domain. Locations of N and C lobes of N-terminal loop are highlighted by yellow and scarlet rectangles. **d** N lobe of N-terminal loop binding through interaction with TM1a, and TM5. **e** C-lobe bound to TM8 extension and CTD. **f** N-terminal loop binding narrows down the inward-open entry. Permeation path calculated by HOLE.^12^ Left: green dots represent the permeation path of KCC3 with N-terminal loop. Right: The pore radii along the conducting passage. The result of HOLE is based on coordinates of Y232. **g** DIOA locates in the central cleft. The density is well fit for DIOA. **h** Hydrophobic interaction from TM10, scissor helices and TM12’ stabilizes DIOA. **i** Hydrophilic interaction from TM10 and TM12. The interaction with R617 of the TM10–11 loop stabilizes the conformation of TM10. **j** Conformational change induced by DIOA binding. The alignment takes the reference of one the scissor helices as in which binds DIOA. Left panel: DIOA induce conformational change of R617 and TM10. Right panel: the rotation of TMD results in the enlarged distance between CTD and of N-terminal loop.

A unique “L” shape N-terminal loop (residues N100–N120) is bound to the entry of the cytosolic vestibule of the transporter (Fig. 1c), which can be divided into an N lobe and a C lobe. The N lobe is anchored between the TM1, TM5, and intracellular loop between TM2 and TM3 (IL23) through electrostatic interactions (Fig. 1d). The C lobe, which extends along TM8 and approaches CTD, is stabilized through salt bridges E116-K549 and E119-R886, as well as hydrophilic interaction between H115 and F203 from IL23 (Fig. 1e). The corresponding N-terminal loop of KCC2 (residues A65-N83) is also bound in the same location with the same mode as KCC3, consist with the sequence conservation in this region (Supplementary information, Fig. S4a).

The binding locks the transporter in inward-facing state, prohibits the conformation change of the transporter. At the same time, it seals up the cytosolic entry (Fig. 1f). Many disease-related mutations of the SLC12 family members are mapped around this region^6,7^ (http://www.hgmd.cf.ac.uk) (Supplementary information, Fig. S4b), highlighting its importance. To further investigate the regulation mechanism of the binding of N-terminal loop, we designed an N-terminal loop deletion mutant, which exhibited higher transport activity than WT, supporting its inhibitory effect^8^ (Supplementary information, Fig. S4c).

DIOA is an indanyl acetic acid derivative, the inhibitory effect of which was observed in several studies with an estimated IC50 of 13.4 μM.^9^ In order to investigate the inhibition mechanism of DIOA to KCCs, we solved the structure of KCC3 in complex with DIOA at 2.7 Å resolution, in which two non-protein densities are observed in the central cleft and each is built as a DIOA molecule as the density shape is well fit (Fig. 1g). KCC3 is captured in the inward-open state bound with DIOA. The indanyl group of DIOA is surrounded by the hydrophobic residues from TM10, the scissor helix and TM12 of the other protomer (Fig. 1h), while the carbonyl group and the carboxyl group of DIOA interact with K664 in TM12 and a conserved residue R617 in the intracellular loop between TM10 and TM11 (IL_10-11_), respectively (Fig. 1i; Supplementary information, Fig. S5a). Compared with the outward-open conformation APC family protein AdiC,^10^ rotation was observed in the TM domain and the IL_10-11_ (Supplementary information, Fig. S5b). The binding of DIOA can impede the conformational transition by locking the rotation of TM domain and/or the IL_10-11_ loop, resulting in the inhibition of the transport activity (Supplementary information, Fig. S5c). The inhibitory effect of DIOA is shared in both NKCCs and KCCs.^11^ To further explore the inhibition mechanism of DIOA, mutants of R617G and K664I were designed and their transport activity was tested. The R617G mutation increases the transport activity, suggesting the inhibitory effect of IL_10-11_. The K664I mutation induces no significant change (Supplementary information, Fig. S5d). Both mutants designed to abolish DIOA binding are still inhibited by DIOA (Supplementary information, Fig. S5d), indicating the inhibitory effect of the indanyl group in DIOA. There are two conserved negatively charged residues E695 and E696 in the region near scissor helix, which are near R617. To investigate the role of E695 and E696, we generated the E695A and E696A mutations, which reduced the transport activity as shown by the transport assays (Supplementary information, Fig. S5e). These results indicate that E695 (E696) and R617 affect the transport activity via different modes. Multiple disease-related mutations are observed in this region (Supplementary information, Fig. S5f), highlighting the structure integrity for transport activity. DIOA induces conformational change of R617 (Fig. 1j, left). Compared with DIOA absent structure, a small but significant rotation of TMD can be detected in DIOA-bound KCC3 when both structures are aligned to one of the scissor helices. This rotation enlarges the distance between CTD and TMD (Fig. 1j, right), resulting in a loosened coordination of N-terminal loop.

In conclusion, we solved the cryo-EM structures of KCC2 and KCC3, which display a distinct configuration compared to NKCC1. The N-terminal loop of KCC2/3 binds to the intracellular vestibule of the transporter, arresting the transporters in an inhibited state. The complex structure of KCC3 with DIOA reveals that DIOA locks KCC3 in an inward-facing conformation by binding to central cleft of transporter and interacting with IL_10-11_ loop. Together, the structures of KCC2 and KCC3 establish a foundation for the study of KCC structure–function relationships and the design of novel therapeutic modulators.

## Supplementary information

Supplemental material for “Cryo-EM structures of the full-length human KCC2 and KCC3 cation-chloride cotransporters”.
