# Supplementary material for: Cryo-EM structures of the full-length human KCC2 and KCC3 cation-chloride cotransporters
Source: Cell Res. 2020 Nov 16;31(4):482–4. doi: 10.1038/s41422-020-00437-x (PMC8182806; doi:10.1038/s41422-020-00437-x)
Supplement: Supplementary file 1 — Supplemental material for “Cryo-EM structures of the full-length human KCC2 and KCC3 cation-chloride cotransporters”. [file 41422_2020_437_MOESM1_ESM.pdf]

Methods

Expression and purification of KCC2 and KCC3

The protein expression and purification process of the proteins are alike. The human full length KCC2b was purchased from Jingmai Biotech Inc. KCC3b sequence were amplified from cDNA library. All protein encoding sequences were subcloned to pCAG vector with N-terminal FLAG-tag for expression in HEK293F cell lines transfected by polyethylenimines (PEIs, Polysciences). Approximately 1.5 mg plasmids were incubated with 3 mg PEIs in 50 mL SMM 293T-I medium (Sino Biological Inc.) for 30 min before transfection. Cells were cultured in Multitron-Pro shaker (Infors, 130 rpm) under 37℃ for 48hs before collection and resuspension in Lysis Buffer (25 mM Tris 8.0 and 150 mM NaCl). Protease inhibitors containing 1.3 μg/ml aprotinin, 5 μg/ml leupeptin, 1 μg/ml pepstatin (Amresco), and 0.2 mM PMSF (Sigma) were added before cruel-extraction at 4℃ in 1.5% DDM and 0.3% CHS (Anatrace) for 2 hours. After centrifugation at ~18000g for 1 hour, the supernatant was applied to anti-FLAG M2 affinity gel (Sigma). Then washed by Wash buffer which is made up of Lysis buffer supplied with protease inhibitors and 0.02% GDN (Anatrace), the protein was eluted by 0.2mg/ml FLAG peptide. Finally, the protein was concentrated by 50-kDa cut-off Centricon (Millipore) and performed size exclusion chromatography (SEC, Superose 6, 10/300, GE Healthcare) in Wash buffer. The peak fractions were confirmed through SDS-PAGE analysis and concentrated for Cryo-EM sample preparation.

Cell-based assay

KCC3 N-deletion, which is deleted 21 amino acids from 100th to 120th of KCC3b sequence, and single-point mutation constructs in this study were accomplished by standard two-step PCR and verified by sequencing. FluxOR II Green Potassium Ion Channel Assay kit (Invitrogen) is applied to estimate the transport activity of KCC3 WT and the indicated mutant proteins. Cells of SF9 were transfected at 2×10^6 cell/mL by baculovirus as indicated. After 48 hours suspension culturing, cells were placed in plate for 2 hours allowing adhesion. 1 × Loading buffer replaced medium and incubated with the cells in 27 °C for 1 hour. After treatment by low-osmotic buffer (88 mM NaCl, 42 mM NMDG-Cl, 5 mM KCl, 2 mM $CaCl_2$, 1 mM $MgCl_2$, 0.77 mg/ml Probenecid, 10 mM HEPES, pH 7.4, osmolarity 260 mOsm/kg) for 45 min, cells were incubated in Assay Buffer ready for fluorescence measurement by Varioskan LUX microplate reader (Thermo Fisher). For measuring the effect of DIOA on transport activity, 100 μM DIOA was added during low-osmotic treatment. The excitation wave length was ~488 nm with width of 12 nm, and emission at 525 nm was recorded. The average of first 120-130 s measurement act as the baseline. Data was recorded 10 seconds after addition of Basal Potassium Stimulus buffer, and recorded every 2 or 3 s for about 500 seconds if not indicated in the figure legends.

Cryo-EM sample preparation and data acquisition

To prepare cryo-EM samples, aliquots (3 μL) of the purified protein concentrated to ~ 10 mg/mL were placed on glow-discharged holey carbon grids (Quantifoil Au R1.2/1.3). For KCC3-DIOA samples, 500 μM DIOA was incubated with the protein solution for 2 hs before concentrated for cryo-EM sample preparation. The grids were blotted for 5 s and flash-frozen in liquid ethane cooled by liquid nitrogen with Vitrobot (Mark IV, Thermo Fisher Scientific). The prepared grids were transferred to a Titan Krios operating at 300 kV equipped with Gatan K3 detector and GIF Quantum energy filter. Movie stacks were automatically collected using AutoEMation[1] with a slit width of 20 eV on the energy filter and a defocus range from -2.2 μm to -1.2 μm in super-resolution mode at a nominal magnification of 81,000×. Each stack was exposed for 2.56 s with an exposure time of 0.08 s per frame, resulting in a total of 32 frames per stack. The total dose rate was approximately 50 $e^-/Å^2$ for each stack. The stacks were motion corrected with MotionCor2[2] and binned 2-fold, resulting in a pixel size of 1.087 Å/pixel. Meanwhile, dose weighting[3] was performed. The defocus values were estimated with Gctf[4].

Data processing

Cryo-EM data was processed similarly for all protein samples. Particles were automatically picked using Relion 3[5] from manually selected micrographs. After 2D classification, good particles were selected and subjected to global angular searching 3D classification against an initial model generated with Relion 3 with C2 symmetry. For each of the last several iterations of the global angular searching 3D classification, a local angular searching 3D classification was performed, during which the particles were classified into 4 classes. Non-redundant good particles were selected from the local angular searching 3D classification. Then, these selected particles were subjected to multi-reference 3D classification, local defocus correction[4], 3D auto-refinement and post-processing. To further improve the map quality, the dataset was classified with adapted masks applied on the intracellular domain (CTD) and the TM/ECD, respectively. For the TM/ECD region, the dataset was C2 symmetry-expanded. The particles with N-terminal loop bound or the particles with well-ordered PHS (phosphorylation-harboring sites, the region around T940 and T997) were selected and further focused refined with appropriate mask.

The 2D classification, 3D classification and auto-refinement were performed with Relion 3. The resolution was estimated with the gold-standard Fourier shell correlation 0.143 criterion[6,7] with high-resolution noise substitution[8]. Refer to Extended data Fig. 1-2 and Table 1 for details of the data collection and processing.

Model building and structure refinement

Model building of KCC3 was performed ab initio with Phenix[9] and Coot[10] based on the focused-refined cryo-EM maps with aromatic residues as land markers, as most of these residues were clearly visible in our cryo-EM map. Each residue was manually checked with the chemical properties considered during model building. Several segments of the sequence were not modeled because of the invisibility of the corresponding density in the map. The model building of KCC2 was accomplished with similar methods using the KCC3 model as a starting template. Chainsaw in CCP4[11] package was used to substitute the sequence during the model building of KCC2.

Structure refinement was performed with Phenix[9] with secondary structure and geometry restraints to prevent structure overfitting. To monitor the overfitting of the model, the model was refined against one of the two independent half maps from the gold-standard 3D refinement approach. Then, the refined model was tested against the other map[12]. Statistics associated with data collection, 3D reconstruction and model building can be found in Table S1.

Table S1 | Data collection, 3D reconstruction and model statistic

**Data collection**			
EM equipment	Titan Krios (Thermo Fisher Scientific)		
Voltage (kV)	300		
Detector	Gatan K3 Summit		
Energy filter	Gatan GIF Quantum, 20 eV slit		
Pixel size (Å)	1.087		
Electron dose (e/Å^2)	50		
Defocus range (µm)	-1.2 ~ -2.2		
Sample	KCC3	KCC2	KCC3-DIOA
Number of collected micrographs	4,488	2,184	3,460
Number of selected micrographs	3,783	1,862	3,201
**3D Reconstruction**			
Software	Relion 3.0		
Number of used particles (Overall)	453,155	186,236	364,959
Resolution (Å)			
Overall	3.3	3.2	2.7
TM_Extracellular	3.1	2.9	2.7
Intracellular	2.9	2.9	2.8
Symmetry	Overall: C2; TM_Extracellular: C1; Intracellular: C2		
Map sharpening B-factor (Å^2)	Overall: -90; TM_Extracellular: -150; Intracellular: -90		
**Refinement**			
Software	Phenix		
Cell dimensions			
a=b=c (Å)	347.84		
α=β=γ (˚)	90		
Model composition			
Protein residues	1,876	1,872	1770
Side chains assigned	1,876	1,850	1770
Sugar	16	14	16
K+	2	2	2
Cl-	4	2	4
inhibitor			2
Water	2	2	40
R.m.s deviations			
Bonds length (Å)	0.005	0.009	0.006
Bonds Angle (˚)	0.967	1.155	1.006
Ramachandran plot statistics (%)			
Preferred	89.29	88.19	91.75
Allowed	10.28	10.94	7.91
Outlier	0.43	0.87	0.34

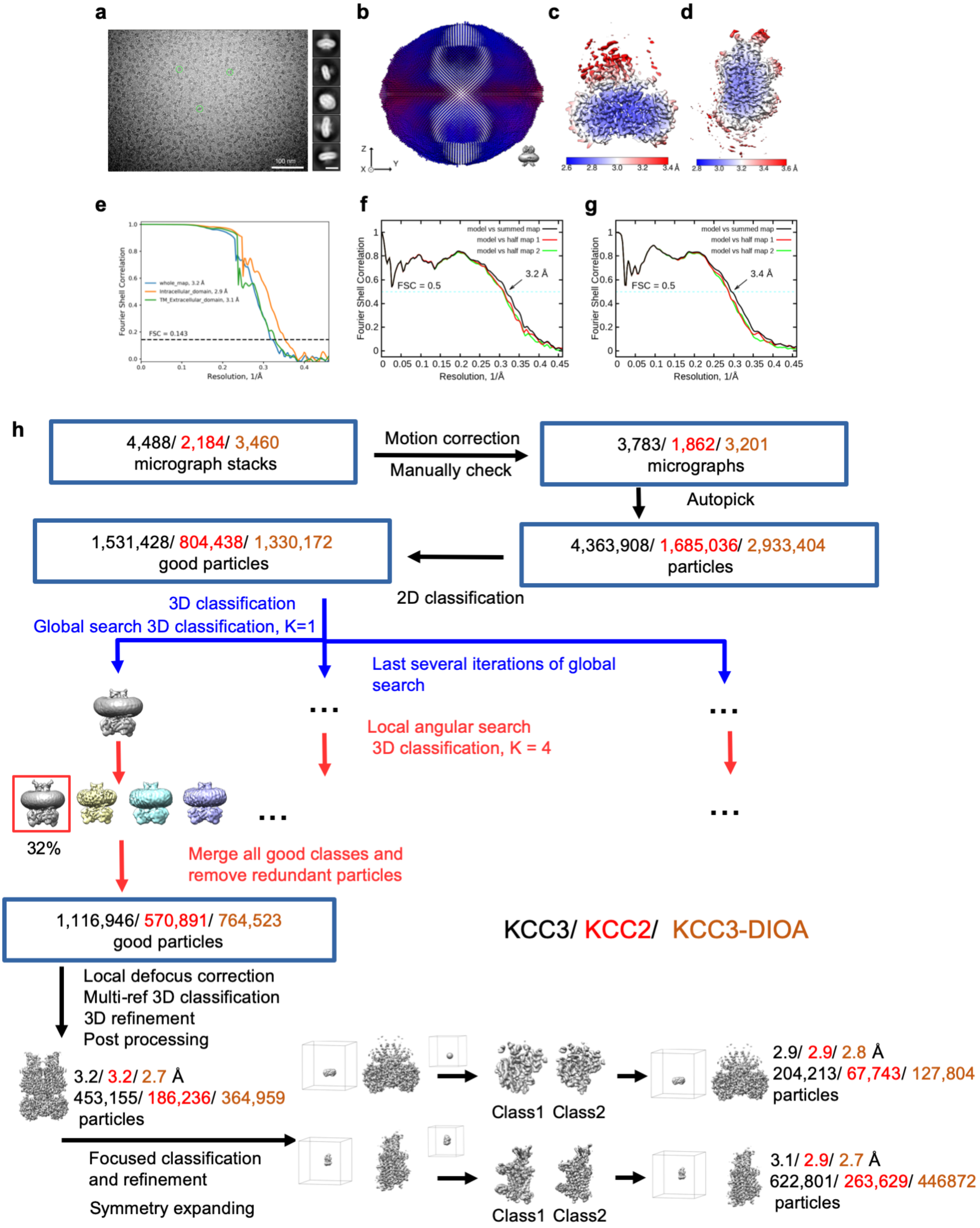

Fig. S1 Cryo-EM analysis of KCC3, KCC2, and KCC3-DIOA.

a Representative Cryo-EM micrograph of KCC3 with results of 2D-class average shown in the left.

b Euler angle distribution of KCC3.

c and **d** Local resolution maps for the 3D reconstruction of KCC3 focused on the intracellular domain or TM region and extracellular domain, respectively.

e Gold standard FSC curve for the 3D refinement of the overall structure, and the 3D refinements focused on the intracellular domain and TM region and extracellular domain, respectively.

f FSC curve of the refined model of KCC3 versus the intracellular domain map that it is refined against [13]; FSC curve of the model refined against the first half map versus the same map (red); and FSC curve of the model refined against the first half map versus the second half map (green). The small difference between the red and green curves indicates that the refinement of the atomic coordinates did not suffer from overfitting.

g is same to f, but for the TM/ECD region of KCC3.

h Flowchart for cryo-EM data processing. Please refer to the 'Data Processing' in Methods section for more details.

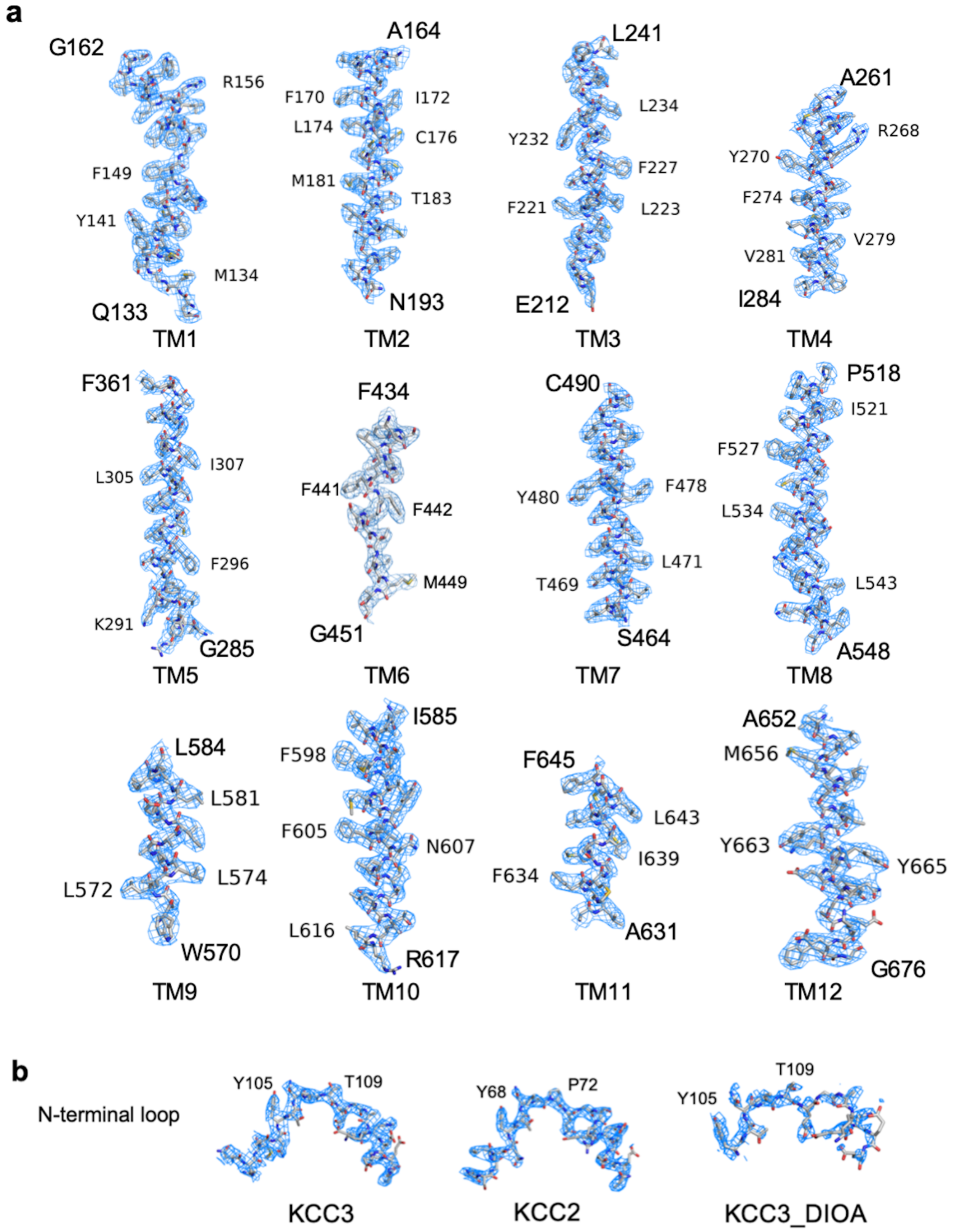

Fig. S2 Cryo EM density maps.

a Cryo EM density maps for TM segments of KCC3 shown at threshold of 8 σ. The amino-acid in the both ends of the TMs are labeled with enlarged characters.

b Cryo EM density maps of N-terminal loop shown at threshold of 12 σ.

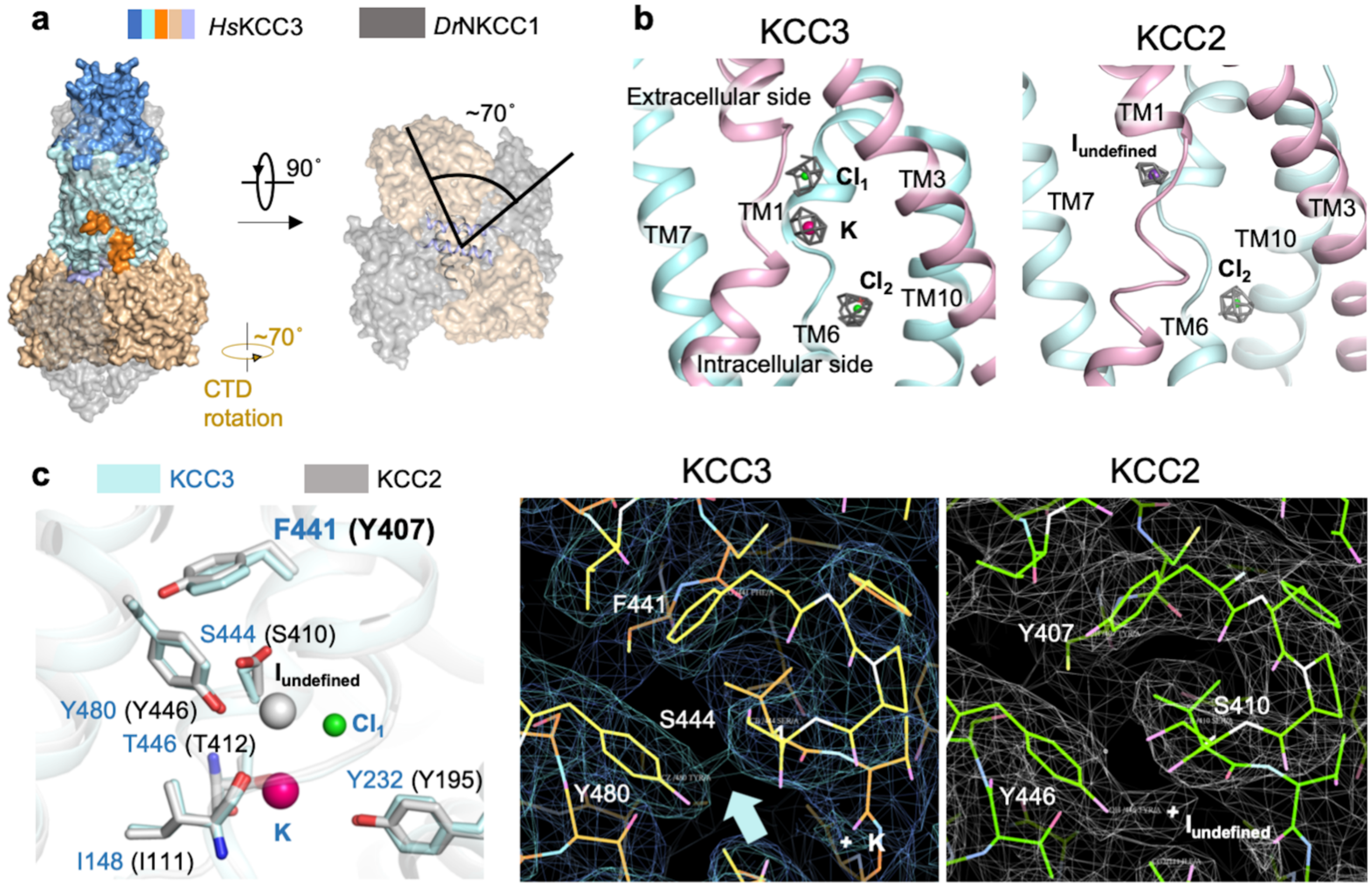

Fig. S3 Domain arrangement of KCC3 and ion binding sites in KCC2.

a Structure compare with NKCC1. The CTD of KCC3 rotate ~70º compared with NKCC1 when both are aligned to the TMD. The TMD and ECD are hided in right panel for a better view of scissor helices.

b Ion binding in KCC3 and KCC2. Residues incorporation of ions mostly are from TM1, 3, 6, 7 and 10. The unknown ion binding in KCC2 are labeled as $I_{undefined}$.

c The amino acid substitution of tyrosine to phenylamine (F441) in KCC3 (the bold label) reduces the attraction of the nearby S444. Then possible link between S444 and Y480 is available (as pointed out by the arrow, middle), which hampers the coordination of $I_{undefined}$.

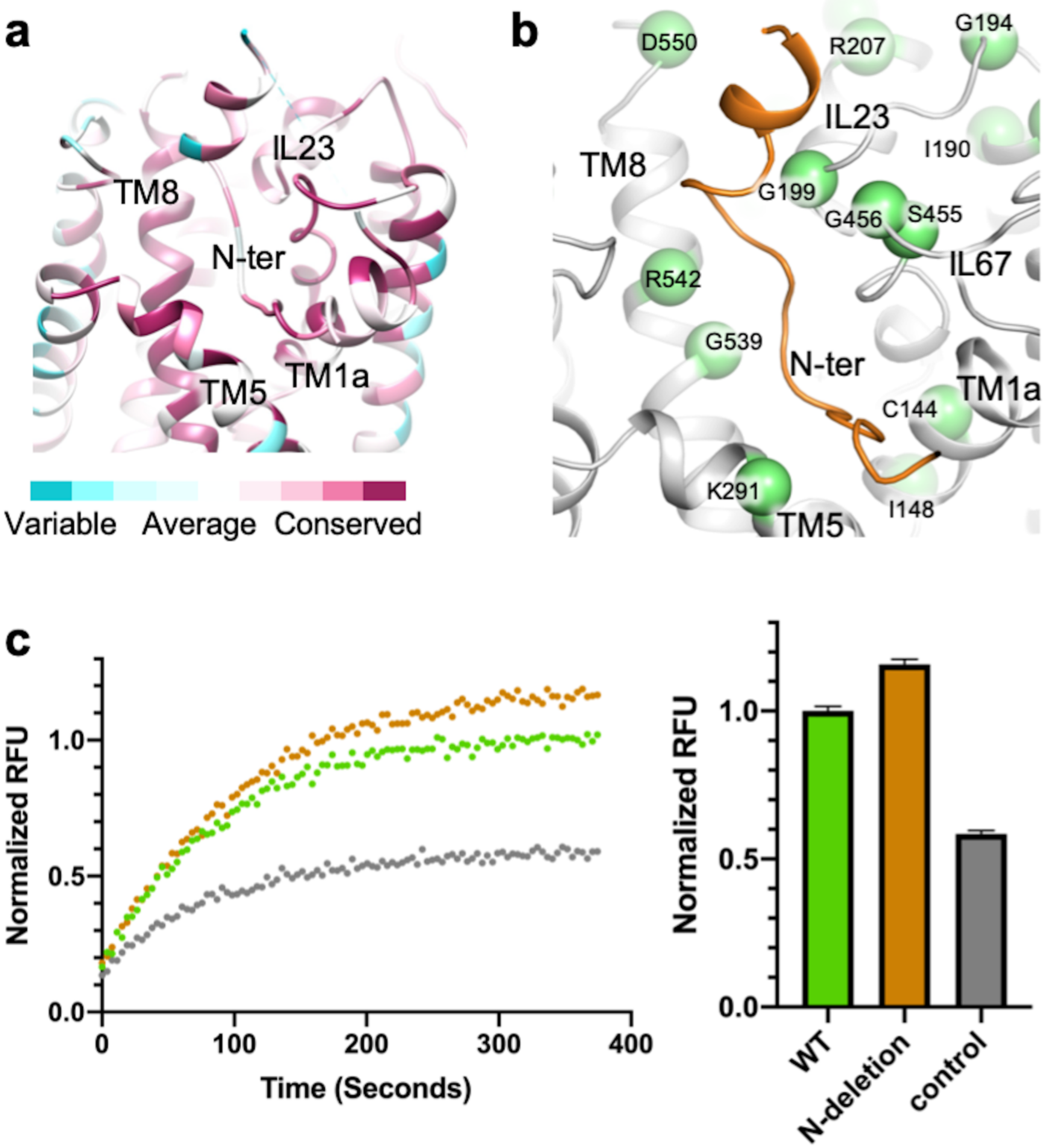

Fig. S4 N-terminal loop binding in KCC3.

a The conservation results around N-terminal loop calculated by Consurf [14,15]. The high conservation regions are labeled in red, while region with low conservation are labeled in cyan. N-deletion stands for N-terminal loop deletion of KCC3.

b Disease-related mutations of SLC12 family members are mapped to KCC3 around N-terminal binding region. The data was derived from the Human Gene Mutation Database (http://www.hgmd.cf.ac.uk/ac/index.php) and two related reviews[16,17]. Green spheres mark the Cα of the disease mutation residues.

c N-terminal loop deletion (N-deletion) has higher transport activity than Wild Type. The fluorescence readout is plotted after normalization against the average of the last 20 readouts of Wild Type (Orange for N-

deletion, Green for Wild Type, Grey for control, n=3 for every group). Data was recorded 10 seconds after addition of Basal Potassium Stimulus buffer, and recorded every 3 s for about 375 s, then every 10 s for 230 s. The average of last 20 measurements are plotted for each group. All other groups are statistic differences with Wild Type when estimated by two-way Anova ($p<0.0001$). Control stands for un-transfected SF9 cells.

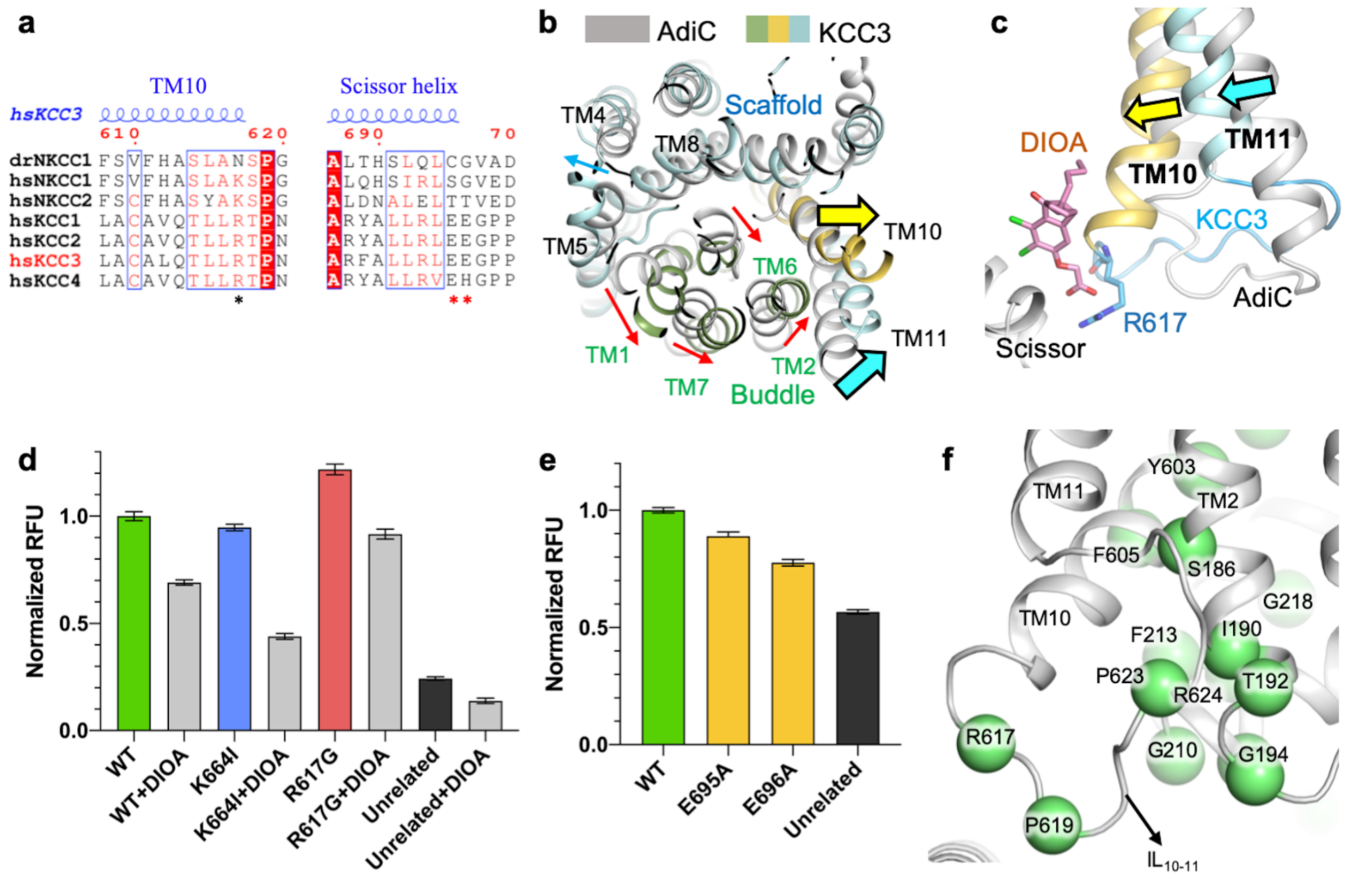

Fig. S5 The inhibitory effect of DIOA.

a Sequence alignment results around R617, E695, and E696. Secondary structures are labeled according to KCC3. Amino acid numbered after KCC3. hs: *Homo sapiens*, Dr: *Danio rerio*.

b Structure comparison between KCC3 and outward-open AdiC (PDB ID: 3H5M). Outlying movement relative the transporter center was observed both in Bundle and Scaffold domains. rotation of TM10 and TM11 are highlighted.

c DIOA induced stabilization of TM10-11 loop hampers the possible conformation change of TM10 and TM11.

d Mutations of KCC3 R617 increase transport activity. The measurement continued for around 600 seconds. The fluorescence readout is normalized against the average of the last 30 readouts of Wild Type. Unrelated stands for transfection of an unrelated baculovirus. n=3 for all groups. Statistic differences with WT are estimated by two-way Anova ($p<0.0001$), as different batches of cell are used. R617G show significant increase while Unrelated showed significant decrease compared with WT. DIOA retains the inhibitory effect of the mutants.

e E695A and E696A in the post-scissor loop mildly influence the transport activity. The fluorescence readout is normalized against the average of the last 30 readout of Wild Type (WT). Green for Wild Type, light blue for E695A, pink for E696A, dark grey for unrelated baculovirus. n=3 for all groups. The average of last 30 measurements of each group are normalized against that of WT. Significant statistic differences are observed between WT and other groups, estimated by two-way Anova ($p<0.0001$), as different batches of cells were

applied.

f Disease mutations mapped from SLC12 family members are labeled by green sphere in Cα. Mutations data is from the Human Gene Mutation Database (http://www.hgmd.cf.ac.uk/ac/index.php) and two related reviews[16,17].

References

1 Lei, J. & Frank, J. Automated acquisition of cryo-electron micrographs for single particle reconstruction on an FEI Tecnai electron microscope. *J Struct Biol* **150**, 69-80, doi:10.1016/j.jsb.2005.01.002 (2005).

2 Zheng, S. Q. *et al.* MotionCor2: anisotropic correction of beam-induced motion for improved cryo-electron microscopy. *Nat Methods* **14**, 331-332, doi:10.1038/nmeth.4193 (2017).

3 Grant, T. & Grigorieff, N. Measuring the optimal exposure for single particle cryo-EM using a 2.6 A reconstruction of rotavirus VP6. *Elife* **4**, e06980, doi:10.7554/eLife.06980 (2015).

4 Zhang, K. Gctf: Real-time CTF determination and correction. *J Struct Biol* **193**, 1-12, doi:10.1016/j.jsb.2015.11.003 (2016).

5 Zivanov, J. *et al.* New tools for automated high-resolution cryo-EM structure determination in RELION-3. *Elife* **7**, doi:10.7554/eLife.42166 (2018).

6 Rosenthal, P. B. & Henderson, R. Optimal determination of particle orientation, absolute hand, and contrast loss in single-particle electron cryomicroscopy. *J Mol Biol* **333**, 721-745 (2003).

7 Scheres, S. H. & Chen, S. in *Nature methods* Vol. 9 853-854 (2012).

8 Chen, S. *et al.* High-resolution noise substitution to measure overfitting and validate resolution in 3D structure determination by single particle electron cryomicroscopy. *Ultramicroscopy* **135**, 24-35, doi:10.1016/j.ultramic.2013.06.004 (2013).

9 Adams, P. D. *et al.* PHENIX: a comprehensive Python-based system for macromolecular structure solution. *Acta Crystallogr D Biol Crystallogr* **66**, 213-221, doi:10.1107/S0907444909052925 (2010).

10 Emsley, P., Lohkamp, B., Scott, W. G. & Cowtan, K. Features and development of Coot. *Acta Crystallogr D Biol Crystallogr* **66**, 486-501, doi:10.1107/S0907444910007493 (2010).

11 Collaborative Computational Project, N. The CCP4 suite: programs for protein crystallography. *Acta Crystallogr D Biol Crystallogr* **50**, 760-763, doi:10.1107/S0907444994003112 (1994).

12 Amunts, A. *et al.* Structure of the yeast mitochondrial large ribosomal subunit. *Science (New York, N.Y.)* **343**, 1485-1489, doi:10.1126/science.1249410 (2014).

13 Fruen, B. R. *et al.* Regulation of the RYR1 and RYR2 Ca2+ release channel isoforms by Ca2+-insensitive mutants of calmodulin. *Biochemistry* **42**, 2740-2747, doi:10.1021/bi0267689 (2003).

14 Caron, L., Rousseau, F., Gagnon, E. & Isenring, P. Cloning and functional characterization of a cation-Cl- cotransporter-interacting protein. *J Biol Chem* **275**, 32027-32036, doi:10.1074/jbc.M000108200 (2000).

15 Glaser, F. *et al.* ConSurf: identification of functional regions in proteins by surface-mapping of phylogenetic information. *Bioinformatics* **19**, 163-164, doi:10.1093/bioinformatics/19.1.163 (2003).

16 Duy, P. Q., David, W. B. & Kahle, K. T. Identification of KCC2 Mutations in Human Epilepsy Suggests Strategies for Therapeutic Transporter Modulation. *Front Cell Neurosci* **13**, 515, doi:10.3389/fncel.2019.00515 (2019).

17 Vargas-Poussou, R. *et al.* Spectrum of mutations in Gitelman syndrome. *J Am Soc Nephrol* **22**, 693-703, doi:10.1681/ASN.2010090907 (2011).
